# Differential patterns of nitrogen nutrition and growth cost of the indigenous *Vachellia sieberiana* and the introduced *Chromolaena odorata* in the savannah environment

**DOI:** 10.1093/aobpla/plz008

**Published:** 2019-02-27

**Authors:** Zimbini Ndzwanana, Zivanai Tsvuura, Alex J Valentine, María A Pérez-Fernández, Anathi Magadlela

**Affiliations:** 1 School of Life Sciences, University of KwaZulu-Natal, Scottsville, Pietermaritzburg, South Africa; 2 Botany and Zoology Department, University of Stellenbosch, Matieland, Stellenbosch, South Africa; 3 Ecology Area, University Pablo de Olavide, Sevilla, Spain

**Keywords:** *Chromolaena odorata*, competition, N_2_ fixation, phosphorus (P) deficiency, savanna ecosystem, *Vachellia sieberiana*

## Abstract

*Vachellia sieberiana* fixes atmospheric nitrogen (N) and distributes it back into ecosystems. We hypothesize that biological nitrogen fixation in this plant species is limited by competition from the invasive shrub, *Chromolaena odorata*. Competition would therefore result in the legume plant switching its limited nitrogen (N) sources in phosphorus-poor soils in savannah ecosystems when resources have to be shared. This study investigated the different patterns of N use and growth costs by a native and an introduced leguminous shrubby species. We propose that the two species sharing the same environment might result in competition. The competitive effect would induce in the indigenous legume to better utilize atmospheric-derived N modifying plant growth kinetics and plant mineral concentrations. Seedlings of *V. sieberiana* were cultivated in natural soil inoculum with low levels of phosphorus (mg L^−1^ ± SE) of 3.67 ± 0.88. The experiments were divided into two treatments where (i) seedlings of *V. sieberiana* were subjected to competition by cultivating them together with seedlings of *C. odorata*, and (ii) seedlings of *V. sieberiana* were cultivated independently. Although *V. sieberiana* was subjected to competition, the N_2_-fixing bacteria that occupied the nodules was *Mesorhizobium* species, similar to plants not subjected to competition. Total plant biomass was similar between treatments although *V. sieberiana* plants subjected to competition accumulated more below-ground biomass and showed higher carbon construction costs than plants growing individually. Total plant phosphorus and nitrogen decreased in seedlings of *V. sieberiana* under competition, whereas no differences were observed in percent N derived from the atmosphere (%NDFA) between treatments. The specific nitrogen utilization rate (SNUR) was higher in *V. sieberiana* plants subjected to competition while specific nitrogen absorption rate (SNAR) showed the opposite response. *Vachellia sieberiana* is highly adapted to nutrient-poor savannah ecosystems and can withstand competition from invasive shrubs by utilizing both atmospheric and soil nitrogen sources.

## Teaser

*Vachellia sieberiana* is a Fynbos legume that is highly adapted to nutrient-poor savannah ecosystems and can withstand competition from invasive shrubs like *Chromolaena odorata* by utilizing both atmospheric and soil nitrogen sources. The shift in the use of N resources is driven by soil-borne symbionts to the plant in the Family Rhizobiaceae, which enhance below-ground allocation to nodules and make them more efficient at fixing biological N. In this way, *V. sieberiana* seedlings subjected to competition have C growth costs when integrated over the whole plant growth cycle compared with *V. sieberiana* seedlings growing with no competition, and allocate more biomass to the below-ground structures that let them survive in impoverished soils.

## Background

The family Leguminosae is the third largest plant family, ranking behind Asteraceae and Orchidaceae and is by far the most widespread group of plants not present only in Antarctica and the Falkland Islands (Polhill *et al.* 1981; Sprent 1995). Legumes are ecologically important because of their ability to fix atmospheric N, thus regulating the input of this nutrient into ecosystems (Zahran 1999; Cramer *et al.* 2010). In ecosystems where N limits plant growth, the presence of legumes greatly influences community dynamics through the provision of fixed N making N limitation a transient condition (Cramer *et al.* 2010; Pirhofer-Walzl *et al.* 2012). The presence of legumes in any ecosystem, including savannahs, is determined among other factors by (i) the energetic requirements of N fixation, (ii) the limitations imposed by other nutrients such as phosphorus and (iii) the ecological factors that include competition, grazing and fire (Crews 1999; Cramer *et al.* 2007). Phosphorus (P) is the second most important nutrient after N and it is a critical limiting factor in legume plants as P deficiency affects both nodule formation and functioning (Graham and Vance 2003; Maseko and Dakora 2013). Nodule formation and functioning is affected by P deficiency through the reduction in the amount of energy available, thereby reducing metabolic rates and the enzyme nitrogenase activity (Tang *et al.* 2001). Furthermore, P deficiency retards plant growth and results in a reduction in the supply of metabolites from the host legume plant to the nitrogen-fixing bacteria; hence, atmospheric N fixation is reduced (Tang *et al.* 2001). Where there is P deficiency, legumes are able to remobilize nitrogen (Cramer *et al.* 2007) to make up for the nutrients deficiency. Given that atmospheric N fixation is an energy intensive process, legumes may use both atmospheric and soil nitrogen sources and the amount of P available in the soil influences such processes (Cramer *et al.* 2007; Abd-Alla *et al.* 2014). The presence of phosphorous scavengers in the same soil where legumes grow might interfere with nodules formation and biological nitrogen fixation (BNF).

The ability of legumes to fix N alters both the patterns of growth and putative competitive outcomes between different plant species through the provision of N to legumes (Thomas and Sumberg 1995). Legumes opting to fix N from the atmosphere instead of taking up N from the soil depend on the available organic and inorganic N and the competition for the available N (Ledgard 2001). Due to atmospheric N fixation, legumes are able to grow alongside or outcompete other plant species while increasing availability of N to plants in the vicinity of the legume (Thomas and Sumberg 1995). Legumes would use atmospheric N while surrounding plants use soil N thereby exerting strong competition for this nutrient which results in insufficient amounts to maintain both the legumes and their competitors (Thomas and Sumberg 1995). Even though legumes may minimize energetic costs associated with atmospheric N fixation by using soil N where there is no competition with other plants, the ability of legumes to fix atmospheric N provides a relative advantage when competition for N is present and when soils are extremely poor in this element (Ledgard 2001). Competition for N is reduced by legumes through their ability to fix atmospheric N and to share that fixed N through various processes (Thomas and Sumberg 1995). Some of these processes include root exudates, connection of roots by mycorrhizal fungi and root contact (Pirhofer-Walzl *et al.* 2012). Legumes are able to fix atmospheric N and potentially share the fixed N provided there is enough P to meet energetic requirements for atmospheric N fixation (Spehn *et al.* 2002).

*Vachellia sieberiana* (formerly known as *Acacia sieberiana*) establishes and grows in nutrient-poor environments that benefit from the legume’s ability to fix atmospheric nitrogen (Otuba and Weih 2015). However, the presence of other species with similar nutritious requirements like might promote changes in the legume preferences for N, inducing sifts from BNF to the use of soil N. *Chromolaena odorata* has invaded the natural ecosystems of *V. sieberiana*, most likely due to its ability to efficiently use the same nutrients as *V. sieberiana* (Mgobozi *et al.* 2008). The displacement of native legumes by invasive plants could be explained by the invasive species’ high growth capacity, enhanced nutrient acquisition rates and resource allocation, which includes P, particularly when available at low concentrations (Dube *et al.* 2017). In South Africa, *C. odorata* is a noxious weed with negative impacts on agricultural lands, grasslands, savannahs and forests (Goodall and Erasmus 1996). *Chromolaena odorata* causes ecological problems in South Africa through ecosystem transformation and ecosystem engineering (Mgobozi *et al.* 2008). Therefore, this species is likely to affect the establishment and growth of the native legume such as *V. sieberiana*, particularly in P-deficient soils.

To our knowledge, no research has been conducted so far to understand how the presence of *C. odorata* affects indigenous legumes in the nutrient impoverished soils of the savanna ecosystems of southern Africa. It has neither being studied the bacterial strains that infect this leguminous species in the used soils from Pietermaritzburg area. To prove that BNF took place we analysed the colour and the content of the nodules of the *V. sieberiana* plant grown in both the control and the treatments. This additionally gives us information on whether the presence of *C. odorata* modifies nodule occupancy in *V. sieberiana*.

This study investigates the competition between *C. odorata* on *V. sieberiana* in nutrient-poor savannah soils and how the presence of the former modifies the patterns of growth of the latter. We examined the effects of competition on the plant biomass accumulation, nodule formation and occupation by nitrogen-fixing bacteria and their subsequent BNF and associated carbon costs. It was hypothesized that (i) *V. sieberiana* subjected to competition with *C. odorata* relies more on atmospheric nitrogen fixation than *V. sieberiana* growing independently and (ii) the *V. sieberiana* seedlings subjected to competition have greater carbon growth costs than *V. sieberiana* seedlings growing with no competitor. As *C. odorata* is an invasive species, we finally draw a line on the implications of its presence to the savannah ecosystems, where *V. sieberiana* is present.

## Materials and Methods

### Plant material and growth conditions

Seeds of *V. sieberiana* were collected from 15 trees in the Pietermaritzburg area prior to starting the experiment. The seeds were scarified by soaking in 95–99 % sulphuric acid (H_2_SO_4_) for 30 min, followed by 10 rinses in distilled water. Seeds were germinated using field-collected soils, which were collected from 20 locations where *C. odorata* and *V. sieberiana* co-occur. The soils were used as the natural source for bacterial inoculum and growth substrate in greenhouse pots (15 cm diameter at top; 10 cm diameter at base; 12 cm height) with perforated bases. All soils were collected from the buffer zone of the Buffelsdraai Landfill (29.631873°S, 30.976778°E) in the province of KwaZulu-Natal, South Africa. These field-collected soils were categorized as nutrient-poor, with P concentrations (mg L^−1^ ± SE) of 3.67 ± 0.88, percentage nitrogen concentration (% ± SE) of 0.17 ± 0.02 and total cations of 7.19 ± 0.20. Cuttings of *C. odorata* collected from the site were grown in a mist house at the University of KwaZulu-Natal’s Botanical Garden in Pietermaritzburg. Lower tips of cuttings were moistened and dipped into Dynaroot rooting hormone powder with 4-indole-3-butyric acid (IBA) as an active ingredient. Thereafter, cuttings were inserted in vermiculite obtained from SA Vermiculite, Johannesburg, South Africa, and grown in the mist house. The experimental design was a complete random block, where pots were randomly rearrange in the glass house every 2 weeks. After 30 days following root formation, cuttings were transplanted into plastic pots containing *V. sieberiana* plants. Each *V. sieberiana* seedling was grown with four cuttings of *C. odorata* from one genotype as neighbours (experiment) or independently grown (control). The design of the study was therefore an additive competition experiment (Goldberg and Scheiner 2001). Ambient conditions in the greenhouse were as follows: night and day temperature range of 12–30 °C, humidity of 70–80 % and an irradiance of 35 %. The experiment and control each had 25 samples. An additional 10 *V. sieberiana* seedlings were harvested at the start of the experiment and used for measurement of initial plant dry weight and N content. Experimental and control plants were grown in the greenhouse for 4 months and were watered when needed by the addition of 500 mL of tap water to each pot.

### Plant harvest and nutrient analysis

Plant harvest occurred 90 days after subjecting *V. sieberiana* to competition from *C. odorata*. The harvested materials were separated into nodules, roots, stems and leaves. Roots, stems and leaves were oven-dried (80 °C, 48 h) and weighed. Nodules were counted per plant and surface sterilized immediately. The sterilization involved the immersion of nodules in 70 % ethanol for 30 s followed by immersion for 180 s in a 3 % sodium hypochlorite solution to remove any microbial life in the outside of the nodules. Nodules were then washed with distilled water five times. Thereafter, nodules were kept in airtight, closed vials that contained silica and cotton wool and were stored at −80 °C before extraction of N_2_-fixing bacteria. The dried plant material was ground using a tissue analyser before analysis for C, N and P concentrations as well as δ^15^N analyses using inductively coupled mass spectrometry (ICP-MS) and a LECO-nitrogen analyser with suitable standards (Central Analytical Facilities, Stellenbosch University and the Archeometry Department, University of Cape Town, South Africa).

### Carbon and nutrition cost and allocation calculations

Carbon construction costs (CW) (mmol C g^−1^ DW), were calculated using the equation of Mortimer *et al.* (2005):

$$\begin{array}{l} CW=[(C+(kN/14)*180/24)*(1/0.89)(6000/180)] \end{array}$$

where CW is the construction cost of the tissue (mmol C g^−1^ DW), C is the carbon concentration (mmol C g^−1^), *k* is the reduction state of the N substrate (*k* = −3 for NH_3_), N is the nitrogen content of the tissue (g^−1^ DW) (Williams *et al.* 1987). The constant 1/0.89 represents the fraction of the carbon construction costs that provides reductant not incorporated into the plant biomass (Peng *et al.* 1993) and (6000/180) converts units of g glucose DW^−2^ to mmol C g^−1^ DW.

Specific nitrogen absorption rate (SNAR) (mg N g^−1^ root DW day^−1^) refers to the net nitrogen absorption rate per unit root DW (Nielsen *et al.* 2001):

$$\begin{array}{ll} SNAR= & \;[\;(N_{2}-N_{1})/(t_{2}-t_{1})\;]\;\\ & *\;[(log_{e}R_{2}-log_{e}R_{1})/(R_{2}-R_{1})] \end{array}$$

where SNAR is the specific nitrogen absorption rate. N_2_ and N_1_ are the final and initial nitrogen content, respectively; *t*_2_ − *t*_1_ is the difference in time between germination and harvesting. *R*_2_ and *R*_1_ are the final and initial root dry weight.

Specific nitrogen utilization rate (SNUR) (g DW mg^−1^ N day^−1^) is a measure of the DW gained for the N taken up by the plant (Nielsen *et al.* 2001):

$$\begin{array}{ll} SNUR=\; & \left[(W_{2}-W_{1})/(t_{2}-t_{1})\right]\\ & *\;[(log_{e}N_{2}-log_{e}N_{1})/(N_{2}-N_{1})] \end{array}$$

where *W*_2_ and *W*_1_ are the final and initial plant dry weights, respectively. N_2_ and N_1_ are the final and initial nitrogen content taken up by the plant, respectively. *t*_2_ and *t*_1_ is the difference in time between germination and harvesting.

Relative growth rate (RGR) values were calculated according to the method proposed by Agren and Franklin (2003).

$$\begin{array}{l} RGR=[(ln\;W_{2}-ln\;W_{1})/(t_{2}-t_{1})] \end{array}$$

where *W*_1_ and *W*_2_ are the dry weights at initial and final harvests, respectively.

### Calculations of the percentage of nitrogen derived from the atmosphere (%NDFA)

δ^15^N analyses were conducted by a commercial laboratory, using inductively coupled mass spectrometry (ICP-MS) and a LECO-nitrogen analyser with suitable standards (Central Analytical Facilities, Stellenbosch University and the Archeometry Department, University of Cape Town, South Africa). Thereafter, percentage N derived from the atmosphere (%NDFA) was calculated. The isotopic ratio of δ^15^N was calculated as δ = 1000 ‰ (*R*_sample_/*R*_standard_), where *R* is the molar ratio of the heavier to the lighter isotope of the samples and standards according to Farquhar *et al.* (1989).

%NDFA was calculated according to Shearer and Kohl (1986):

$$\begin{array}{ll} \%NDFA=\; & 100\;((\delta^{15}N_{reference\;plant}-\delta^{15}N_{legume\;plant})\\ & /(\delta^{15}N_{reference\;plant}-B)) \end{array}$$

where *B* is the δ^15^N natural abundance of the nitrogen derived from BNF of the above-ground tissue of *V. sieberiana* grown in a nitrogen-free culture. The *B* value of *V. sieberiana* was determined in this study as −2.58 ‰.

### Bacterial DNA extraction and sequencing

Presumptive root-nodule-forming and N-fixing bacteria were isolated from the stored nodules (43 nodules were taken from the competition treatment and 17 from the control) and grown on yeast mannitol agar (YMA), incubated at 28 °C. Culture purity was verified by repeated streaking of single-colony isolates. Genomic DNA was extracted using the Quick-gDNA™ MiniPrep kit (Zymo Research). A portion of the 16S rDNA gene was amplified for all the pure bacterial colonies through PCR reactions using the primers: 27F (5′ AGA GTT TGA TCC TGG CTC AG 3′) (Suau *et al.* 1999) and 1492R (5′ GGT TAC CTT GTT ACG ACT T3′) (Kato *et al.* 1997). PCR cycle conditions included a initial denaturation at 95 °C for 5 min, followed by 30 cycles of denaturation at 95 °C for 1 min, annealing at 55 °C for 1 min, extension at 72 °C for 1 min, and a final elongation step of 72 °C for 10 min. The PCR reaction volumes (for a total of 25 μL) were: 16.3 μL sterile distilled water, 2 μL (25 mM) MgCl_2_, 2.5 μL (10×) Buffer, 0.5 μL of (10 μΜ) primer 27F, 10 μL (10 μM) primer 1492R, 2 μL (2.5 mM of each dNTP), 0.2 μL SuperTherm Taq and 1 μL of DNA (50–100 ng).

Enzymatic clean-up of the PCR products was performed using Exonuclease I (0.5 μL), placing the reaction on the heating block at 37 °C for 15 min, thereafter 2 μL FastAP (ThermoFischer Scientific, South Africa) was added and the heating block set higher to 85 °C for another 15 min. To view the results of the clean-up gel electrophoresis was once again used. Thereafter, the cleaned amplicons were included in a sequencing PCR with a cycle of 96 °C for 5 s, 25 cycles of denaturation at 96 °C for 10 s, annealing at 50 °C for 5 s and extension at 60 °C for 4 min 15 s. Each sequencing reaction contained: 2 μL Big Dye solution, 1 μL 5× Sequencing Buffer, 1 μL of the respective primer and 6 μL DNA. The sequencing reaction was only performed in the forward direction (i.e. using the 27F primer) and was done with the ABI PRISM BigDye Terminator v3.0 Cycle Sequencing kit on a ABI 3100 Automated Capillary DNA sequencer (both from Applied Biosystems). Thereafter, the sequencing product was cleaned and precipitated in preparation for Sanger sequencing. To the 10 μL sequencing product 10 μL sterile water, 2 μL 3 M sodium acetate and 50 μL of 100 % ethanol were added. The mixture was incubated on ice for 20 min, thereafter centrifuged for 30 min at 13 000 rpm (at 4 °C). Then the supernatant was removed and 80 μL 70 % ethanol was used to wash the DNA pellet, then centrifuged for 10 min at 13 000 rpm (at 4 °C). The wash step was repeated. The ethanol supernatant was removed and air-dried the DNA pellet for at least 15 min. The samples were then sent to the DNA Sequencing Facility of the University of Pretoria. The resulting chromatograms were subjected to blast searches (National Center for Biotechnology Information (NCBI); https://www.ncbi.nlm.nih.gov) to compare them to all of the other bacterial 16S rRNA sequences already in the database (Kato *et al.* 1997; Suau *et al.* 1999).

### Statistical analysis

Plant performance was measured as biomass accumulation (i.e. root biomass, shoot biomass, total biomass, number of nodules, root-to-shoot ratio, RGR of the plant, RGR of roots, below-ground allocation), P and carbon content, and isotope N and its associated variables (i.e. total plant N concentration, N derived from the atmosphere, % N derived from the soil, % N derived from the atmosphere, SNAR, SNUR). In order to avoid type I errors that may arise from separately testing each dependent variable between the control and experimental treatments multiple times, we used one-way multivariate analysis of variance (MANOVA) (Zar 1999). Three MANOVA tests were carried out, one each for the biomass accumulation variables, nutrient content, and isotope analysis. All analyses were undertaken using IBM SPSS version 25 (IBM SPSS 2017).

## Results

### Biomass and growth kinetics

**Figure 1.**
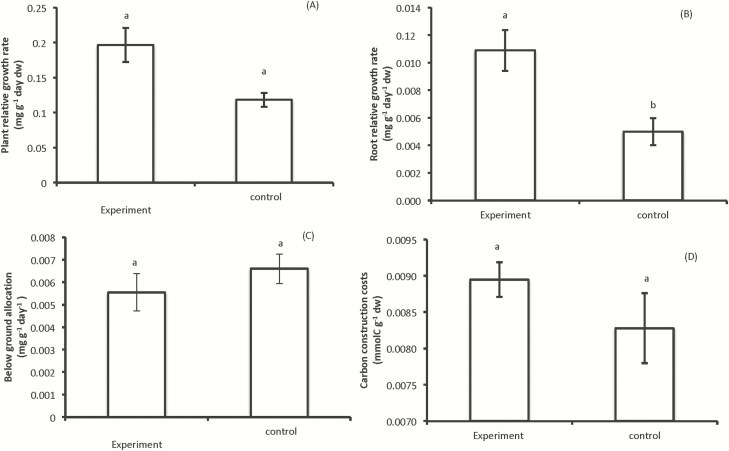
Growth kinetics of 120 days *V. sieberiana* tree legume sharing resources with *C. odorata* and the tree legume, *V. sieberiana* grown independently. Values are means ± 1 SEM. Different letters indicate significant differences among treatments.

Shoot biomass of *V. sieberiana* plants grown singly and those grown with *C. odorata* attained similar values (Table 1). Univariate tests carried out simultaneously as part of the MANOVA analysis showed that total biomass, root biomass and RGR were greater among plants grown with *C. odorata* than those grown singly (Table 1; Fig. 1A). The individual analyses of RGR proved that only the RGR of the roots was statistically significant greater in plants of *V. sieveriana* grown in competition with *C. odorata* were observed for root RGR (Fig. 1B). Total plant biomass of *V. sieberiana* competing with *C. odorata* was 1.57-fold greater than that of plants growing by themselves (Table 1). The greater biomass in plants subject to competition was due to the higher root biomass and nodule numbers than legume plants grown independently (Table 1). No significant difference was detected in below-ground biomass allocation between the legume plants (Fig. 1C; Table 2). The plant RGRs (Fig. 1A) and root:shoot ratio (Table 1) showed the same sequence of results as below-ground biomass allocation.

**Table 1.** Plant biomass, RGR, number of nodules and plant N and P mineral concentrations of 120-day-old *V. sieberiana* tree seedlings grown alone or with *C. odorata*. Values are means ± 1 SEM. Different letters indicate significant differences among treatments.

Trait	*Vachellia* and *Chromolaena*	*Vachellia* alone
Total plant biomass (g)	0.688 ± 0.045a	0.523 ± 0.038b
Shoot biomass (g)	0.236 ± 0.019a	0.267 ± 0.020a
Root biomass (g)	0.452 ± 0.045a	0.257 ± 0.049b
Root-shoot ratio	2.0 ± 0.319a	0.993 ± 0.231a
RGR	0.162 ± 0.012a	0.118 ± 0.010b
Number of nodules	9.0 ± 1.5a	5.3 ± 0.9a
Plant N concentration (mmol g^−1^)	1.31 ± 0.5a	1.79 ± 0.98b
Plant P concentration (mmol g^−1^)	34.49 ± 1.93a	44.14 ± 1.28b

**Table 2.** Pillai’s trace, *F*-ratios and associated probabilities for MANOVA for the biomass accumulation, nutrient content and isotope N variables.

Dependent variables	Pillai’s trace	*F*	df_1_, df_2_	*P*
Biomass accumulation	0.583	0.233	6, 1	0.916
Nutrient content	0.796	7.815	2, 4	0.042
Isotope analysis	1.000	10047	5, 1	0.008

### Carbon construction costs, P and N nutrition

Nitrogen and phosphorous (Table 1) content in plants growing in competition was significantly different in plants grown in the control and in the experiment treatment. This may have been driven by P content of the plants, which was significantly greater in singly grown plants than in those grown with the alien invasive (Table 1). In contrast, legume plants grown with *C. odorata* had insignificantly greater C construction costs than legume plants grown independently (Fig. 1D).

**Figure 2.**
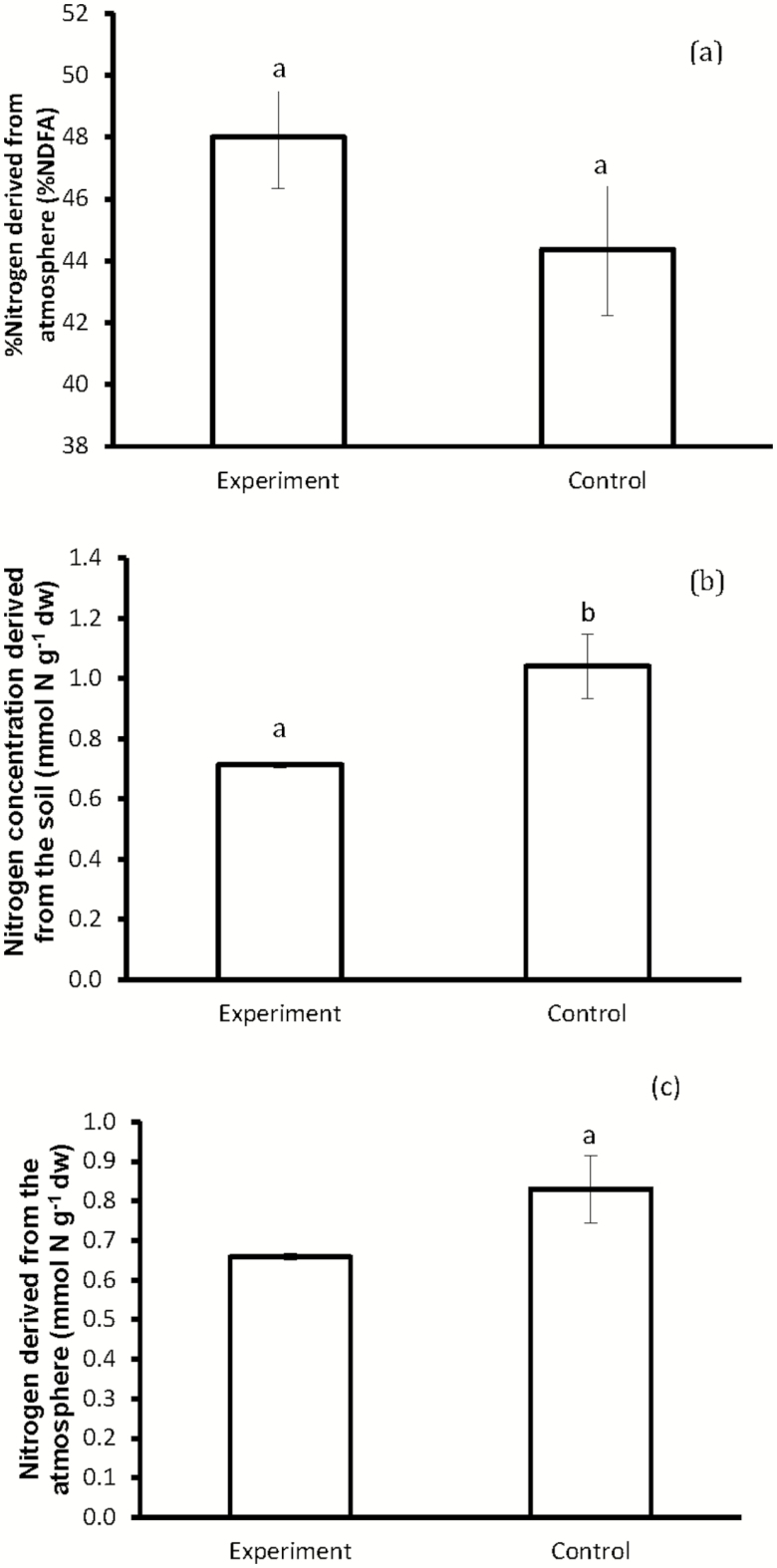
(A) Percentage N derived from the atmosphere, (B) soil N concentration and (C) atmospheric nitrogen concentration of 120 days *V. sieberiana* tree legume grown in the presence of *C. odorata* and the tree legume, *V. sieberiana*, grown independently. Different letters indicate significant differences among treatments.

Legume plants grown in competition showed a higher percentage of N derived from the atmosphere (48 ± 1.7 %) than control plants (44.4 ± 2.1 %), though no statistical significant differences between the treatments were observed (Fig. 2A; Table 2). Total plant N values were low, and were similar between singly grown plants and plants grown under competition (i.e. 1.87 mmol N g^−1^ and 1.37 mmol N g^−1^ dry weight, respectively; Fig. 2B). We note, however, the marginal value of the *P*-value after the statistical analyses (i.e. *F*_1, 5_ = 5.062, *P* = 0.074). Plants growing independently (i.e. control) relied more on both soil N and atmospheric N than plants subject to competition, this is indicated by the total plant N concentration derived from the soil and total plant N concentration derived from the atmosphere (Fig. 2B and C).

**Figure 3.**
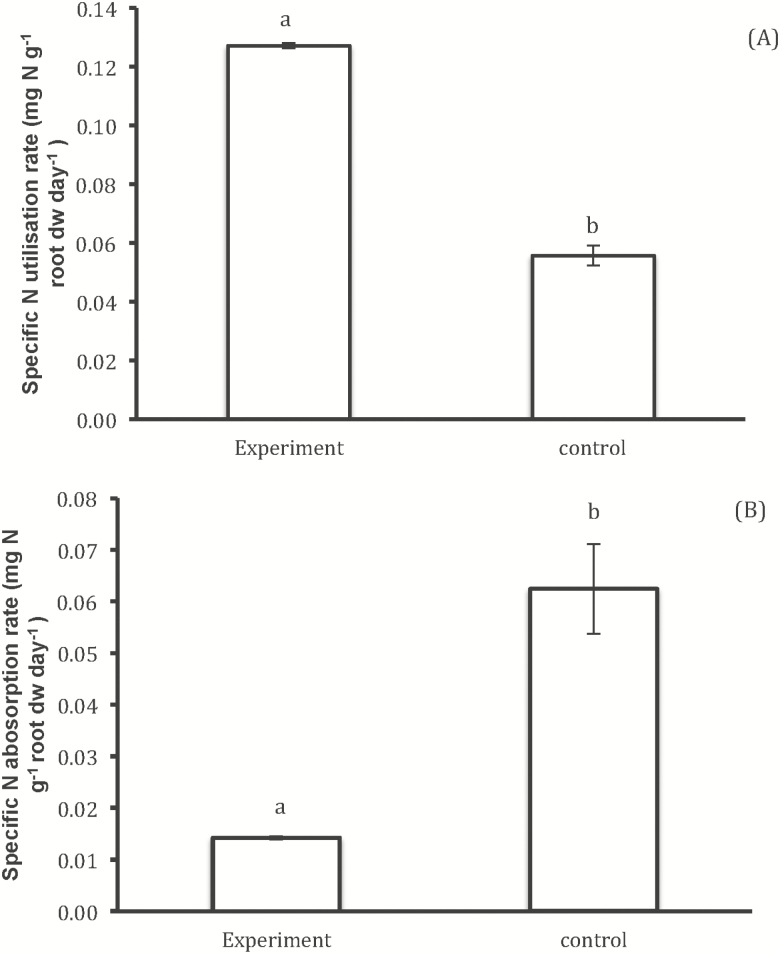
(A) SNAR and (B) SNUR of 120 days *V. sieberiana* tree seedlings subjected to competition with *C. odorata* and the tree legume, *V. sieberiana* grown independently without competition from *C. odorata*. Different letters indicate significant differences.

Furthermore, SNAR was significantly greater in plants growing independently than plants subject to competition, with a value 4-fold greater in the later than in the former (Fig. 3A). Inversely, plants subjected to competition showed greater SNUR than control plants, i.e. 0.127 and 0.056 mg N g^−1^ root DW day^−1^, respectively (Fig. 3B).

### Bacterial species diversity

DNA was successfully isolated from *V. sieberiana* root nodules and amplified using the 16S rRNA bacterial gene. Sequence analysis of the nearly complete 16S rRNA gene amplified revealed that the bacteria represent members of the *Mesorhizobium* sp. potentially nodulate *V. sieberiana.* The results include several sequences with high similarities (90–93 %) to several *Mesorhizobium* sp. (i.e. *Mesorhizoium ciceri* (GenBank accession MF996756.1), *Mesorhizobium plurifarium* (GenBank accession MF996345.1), *Mesorhizobium mediterraneum* (GenBank accession KX959584.1), *Mesorhizobium olivaresii* (GenBank accession NR_149815.1), *Mesorhizonium tianshanense* (GenBank accession KX959571.1)). All bacterial strains were similarly represented in plants from both the control and the experiment treatments.

## Discussion

Our data suggest that *V. sieberiana* plants can overcome nitrogen deficits in the soil shifting from the use of mineral sources of N to BNF. To that end, the plant seems to be rather promiscuous in its selection of symbionts, as it is able to form nitrogen-fixing nodules with a wide range of rhizobia. Plants under stress also increased below-ground biomass, so they can explore a broader area of soil at the time that they have space for a greater number of nodules. This represents a clear advantage for the plant in the poor soils of the South African savannah.

Sequence analysis of the 16S rRNA gene suggests that the N-fixing bacteria housed in the nodules during the legume and invasive plant competition and in control plants remained unchanged for what we cannot conclude that the competition exerts any influence on *V. sieberiana*’s nodule occupancy. Therefore, *V. sieberiana* showed nodulation with a highly effective symbiont with species belonging to the genus *Mesorhizobium*, and maintained nitrogen fixation during competition. The use of 16S rRNA gene sequence analyses allowed efficient identification of the genus *Mesorhizobium* to which *V. sieberiana* root nodule isolates belong, but were not suitable for their identification to the species level. Future studies will therefore seek to identify the legume plant symbionts to species level using other more suitable approaches such as multilocus sequence analysis (MLSA) (Gevers *et al.* 2005). Such results would allow for more detailed examination of the possible bacterial strains housed in the nodules and any changes that might occur during competition evaluated in this study. In spite of this, our sequence data suggest that the bacteria isolated from the nodules must have represented closely related members of the genus *Mesorhizobium.* This is consistent with a study done by Räsänen and Lindström (2003) on drought tolerant and multipurpose African acacia trees which formed nitrogen-fixing nodules with a wide range of rhizobia, including *Mesorhizobium* sp. They further suggested that African acacia species are appropriate trees for reforestation of degraded areas in arid and semiarid regions of the tropics and subtropics as these trees and their symbiotic partners survive harsh growing conditions (Räsänen and Lindström 2003).

**Figure 4.**
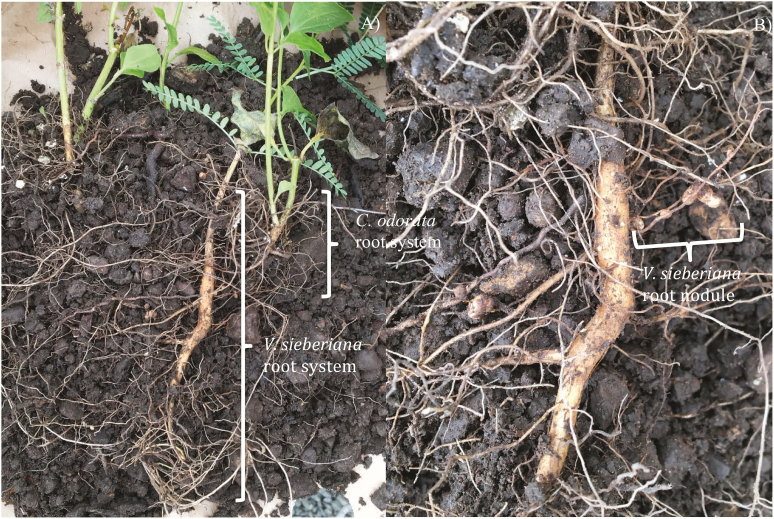
(A) Root systems of *V. sieberiana* tree seedlings and *C. odorata* plants grown together and (B) root nodules of *V. sieberiana* tree seedlings grown independently without *C. odorata*.

The increased below-ground biomass observed in the plants under competition can be seen as a clear need of the plant to search for the same pool of nutrients from the top-soil layer used by the competing plants of *C. odorata* (Cramer *et al.* 2007). The increased competition for nutrients may have resulted in the legume increasing the relative root growth rate to utilize nutrients available at lower soil layers and increase root surface area for nodules as shown in Fig. 4. Legumes reportedly develop adaptations directed to enhance nutrient acquisition and uptake during nutrient deficiency (Horst *et al.* 2001). One of these adaptations is changes in root morphology, forming root clusters to increase root surface area and nodule numbers, these resulting in changes in root to shoot ratio (Lamont 1983; Horst *et al.* 2001; Vance 2001). It is actually known that because of the excessive cost of nodule formation and N-fixing processes, legumes initially try the more energetically advantageous strategy to grow roots to appropriate soil nitrogen (Gutschick 1980). Only when root modifications are not enough to sufficiently acquire N will legumes facilitate nodule formation at the expenses of above-ground biomass production (Vitousek and Howarth 1991). This is consistent with our observations as *V. sieberiana* plants subject to competition had greater root biomass, root RGRs and nodule number. The increased number of nodules as a result of increased root surface explains the legumes’ reliance on atmospheric-derived nitrogen during competition.

*Vachellia sieberiana* increased the number of nodules when subject to competition with *C. odorata* for soil nutrients; hence, competition first induces root size increase and secondly, the formation of nodules that result in increased N-fixing efficiency (Crews 1999). Enhanced nodule efficiency for nutrient utilization is considered a crucial coping strategy during nutrient stress (Høgh-Jensen *et al.* 2002; Vance *et al.* 2003; Mortimer *et al.* 2008; Le Roux *et al.* 2009; Mortime *et al.* 2009). Our results were consistent with this as *V. sieberiana* subject to competition showed more reliance towards atmospheric-derived N than available soil N during growth. The greater efficiency of N fixation may further explain the 48 % N derived from the atmosphere by legume plants subjected to competition compared to 44 % by independently growing plants. N_2_ fixation is assumed to require significantly more energy per N-fixed than combined available soil N uptake and reduction (Gentili and Huss-Danell 2002; Valentine *et al.* 2010). Despite legume plants relying on N derived from the atmosphere with N fixed being 44–48 % of the plant total N, the legume plants also relied on soil N. Both plants exposed to competition and the independently grown plants assimilated soil N, though reliance was observed to be higher in independently grown legume plants resulting in higher N absorption rate (SNAR). This may be due to that the legume plants were exposed to the entire available soil N nutrient, as they were not exposed to soil N nutrient competition with the invasive plant. In contrast, plants exposed to competition showed increased N use efficiency (SNUR). The increased root biomass in legume plants exposed to competition is likely the cause of the increase in the demand for N to maintain the increased root RGRs. This concurs with previous findings (Sa and Israel 1991; Gordon *et al.* 1997; Almeida *et al.* 2000; Magadlela *et al.* 2014) where the decrease in biomass in legume plants caused a reduction in the N demand in the host plant and the inverse was observed in plants with increased biomass. The increase in below-ground biomass and reliance of the legume plants on atmospheric N and soil N during competition and the increased N use efficiency (SNUR) collectively may have resulted in 0.001 (mmol C g^−1^ dw) higher C construction costs (Kaschuk *et al.* 2012). Although differences in C construction costs may be statistically insignificant as they are small when integrated over the whole plant growth cycle, these costs may be significant. During nutrient stress it has been indicated that the below-ground structures required a bit more C per gram of tissue produced, although it is unclear what the precise structural nature of this additional C is, this might be the same during competition (Mortimer *et al.* 2008; Kaschuk *et al.* 2012; Magadlela *et al.* 2014).

Allocation of carbohydrates to various plant parts and functions is a pivotal parameter for plant growth and success during stress conditions (Nielsen *et al.* 1998). In legumes a fraction of the C resources is utilized for construction of plant tissues, and to maintain N_2_-fixing bacterial physiology for effective N fixation (Nielsen *et al.* 1998). The success of plants under stressed conditions, in this regard competition for soil nutrients, may be determined by the plant’s ability to control the C allocation (Nielsen *et al.* 1998). In addition to the increased root growth rates, the cumulative sink effect of the legumes’ nodules might have imposed a drain on the legume plants C reserves to maintain bacterial physiology and N_2_ fixation during competition. This is consistent with findings of Jia *et al.* (2004) in *Vicia faba* and Lynch and Beebe (1995), Nielsen *et al.* (1998) and Mortimer *et al.* (2008) on *Phaseolus vulgaris*, where plants expended a larger C budget on below-ground respiration during nutrient deficiency.

## Conclusion

This study established that *V. sieberiana* grown in competition with *C. odorata* has a shift in its N preference, by relying more on biologically fixed nitrogen compared to plants grown individually. In this way *V. sieberiana* is able to adapt to the competition with *C. odorata*, by increasing its below-ground allocation to nodules and their more efficient function. Furthermore, the results of this study establish that *V. sieberiana* seedlings subjected to competition may have greater C growth costs when integrated over the whole plant growth cycle than *V. sieberiana* seedlings growing with no competitor. These results show that *V. sieberiana* is highly adapted to nutrient-poor savannah ecosystems and can withstand competition from invasive shrubs by increasing below-ground biomass and utilizing both atmospheric and soil nitrogen sources.

## Sources of Funding

This work was funded by the School of Life Sciences at the University of KwaZulu-Natal, Pietermaritzburg and National Research Foundation, South Africa, grant no. 113576 were used for analysis.

## Contributions by the Authors

All authors contributed equally in all steps of the study. All authors read and approved the final manuscript.

## Conflict of Interest

None declared.
